# Endotoxemia is associated with acute coronary syndrome in patients with end stage kidney disease

**DOI:** 10.1186/s12882-017-0652-0

**Published:** 2017-07-12

**Authors:** Chien-Chin Hsu, Tsui-Shan Wei, Chien-Cheng Huang, Yi-Ming Chen

**Affiliations:** 1 Department of Emergency Medicine, Chi-Mei Medical Center, 901, Chung-Hwa Rd, Yongkang District, Tainan, 710 Taiwan; 2 Department of Biotechnology, Southern Taiwan University of Technology, Tainan, Taiwan; 3 Department of Medical Research, Chi Mei Medical Center, Tainan, Taiwan; 4 Department of Environmental and Occupational Health, College of Medicine, National Cheng Kung University, Tainan, Taiwan; 5 Department of Child Care and Education, Southern Taiwan University of Science and Technology, Tainan, Taiwan; 6 Department of Microbiology, College of Medicine, Kaohsiung Medical University, Kaohsiung, Taiwan

## Abstract

**Background:**

Cardiovascular disease is the major cause of death in patients with end-staged kidney disease (ESRD). Most ESRD patients have systemic inflammation, and increasing the risk of cardiovascular event. Endotoxin derived from lipopolysaccharide of Gram negative bacteria accounts for 70% of intestinal bacteria, leading to release of proinflammatory cytokines and negative cardiovascular effect. Impaired intestinal barriers have been found in some ESRD patients, and may lead to bacteria translocation from gastrointestinal tract. We aim to investigate the association of endotoxemia in ESRD patients and acute coronary syndrome (ACS).

**Methods:**

We collected serum from adult ESRD patients who presented to emergency department (ED) with ACS (30 patients) or without ACS (30 patients) as control from 11/01/2013 to 10/31/2014 in Chi Mei Medical Center in southern Taiwan. Clinical information and lab data were collected. We measured the endotoxin level of the serum of ESRD patients with or without ACS. We used real-time 16S rDNA PCR to detect possible bacteria in the blood of the patients.

**Results:**

The endotoxin level of ESRD patients with ACS (0.49 (±0.12) EU/mL) was significantly higher than that of ESRD patients without ACS (0.1 ± 0.08) (*p* < 0.01). However, the endotoxin level was not correlated with the troponin-I level (*r* = −0.12). Although endotoxin level was higher in ESRD patients with ACS, bacteria were not detected in the serum by using the real-time 16S rDNA PCR.

**Conclusion:**

Endotoxin in ESRD patients with ACS was significantly higher than that without ACS. The result suggested that endotoxemia may have a contributory role to cardiovascular disease in ESRD patients.

## Background

Cardiovascular disease is the major cause of death in patients with chronic kidney disease (CKD) [1]. Systemic inflammation increases the risk of cardiovascular disease in the CKD patient [2]. Most of the patients under hemodialysis therapy have systemic inflammation, which is caused in part by catheters, tubes or endotoxin existing in non-ultrapure water used for hemodialysis. However, the source of inflammation is often unknown [3]. New evidence shows that endotoxin and bacteria can be subject to intestinal translocation to the systemic blood stream [4]. Endotoxin is derived from lipopolysaccharide (LPS) which is the major component of the outer membrane of Gram negative bacteria. Gram negative bacteria account for 70% of adult intestinal bacteria. Endotoxin leads to release of proinflammatory cytokines which are connected with CD 14 of immune competent cells, resulting in negative cardiovascular effect, e.g., dilatation of peripheral vessels and impaired cardiac muscle contraction [5].

Intestinal mucosa is the largest physical interface between host and microbes. Diverse microbes exist in the Intestines, consisting over 400 strains of bacteria [4]. One study showed that aerobic (~10^6^ bacteria/ml) and anaerobic (~10^7^ bacteria/ml) bacteria were increased in the duodenum and jejunum in uremic patients; in contrast to healthy individuals, few or no bacteria existed in the area [6]. One animal study showed that uremic rats (8 of 20) were found to have bacterial translocation as compared to normal rats (1 of 20 controls) [7]. Intestinal barriers were impaired in the uremic animal [8] and CKD patients [9]. Ischemic change in the intestinal mucosa was also found in the patients undergoing hemodialysis therapy [10]. Destruction of mucosal structure increased intestinal permeability [11]. Pathological intestinal bacteria, e.g., *Escherichia coli, and Salmonella typhimurium,* produce several kinds of toxins. Microbial translocation in the HIV chronic infected patients has been demonstrated one of the causes in the systemic immune activation [12]. However, the role of microbial translocation in ESRD patients has not been well studied.

Several biomarkers have been used to detect and quantify microbial translocation, for example, LPS and soluble CD14 (sCD14) [13]. The sCD14 ELISA method is applicable, but it is not specific in the detection of microbial translocation, because other cytokines, e.g., INF-α and INF-β may increase CD14 [14, 15]. LPS limulus amoebocyte lysat (LAL) is currently the standard method of detecting endotoxin [16]. However, this method can only detect endotoxin produced by Gram negative bacteria, which is unable to detect microbial translocation of Gram positive bacteria in the intestine. Molecular diagnostics provides an opportunity to detect microbes, particularly viruses or bacteria which are difficult to be cultured, such as tuberculosis or anaerobic bacteria. 16S ribosomal DNA PCR is used to detect the highly-conserved prokaryotic ribosomal RNA gene. Real-time 16S PCR method after eliminating bacterial DNA in the environment can be used to quantify bacteria [17].

We aimed to evaluate the role of microbial translocation in ESRD patients with acute coronary syndrome caused by myocardial injury, contributing to elevated cardiac troponin. We measured troponin-I level, detect endotoxin level and nucleotides of microbes if any in the serum of ESRD patients by ELISA and molecular method.

## Methods

### Blood samples and clinical information of ESRD patients at ED

We collected 60 adult patients (age > 17 years old) with ESRD presented to ED due to acute coronary syndrome (*n* = 30) or without acute coronary syndrome (*n* = 30) as control from 11/01/2013 to 10/31/2014 in one medical center in southern Taiwan. Patients who had hospitalization due to infectious disease within 1 month, or were taking antibiotics were excluded in the study. All patients with ESRD were undergoing hemodialysis. Acute coronary syndrome was defined as clinical symptom of chest pain compatible with unstable angina, non-ST segment elevation myocardial infarction or ST-segment elevation myocardial infarction. Written informed consent for participation in the study was obtained from participants. Serum samples were collected in enotoxin-free glass tubes and stored in −80 °C. Clinical information regarding demographic feature, past history as well as lab data, cardiac sonography, electrocardiography, or coronary angiography (if any) were collected. Clinical information was de-identified. This research project was approved by Institutional Review Board of Chi Mei Medical Center (No. 10212-002).

### Measurement of endotoxin in serum of ESRD patients

Serum samples were numbered and de-identified. We use the method as described previously [12] to measure endotoxin level of patients with ESRD. Serum is diluted with endotoxin-free water to 20% concentration, followed by heating to 70 °C for 10 min to inactivate protein in the serum. We measure and quantify lipopolysaccharide (LPS) level by Limulus Amebocyte assay (Cambrex, Verviers, Belgium) according its protocol. Briefly, a sample was mixed with the LAL supplied in the test kit and incubated at 37 °C (±1 °C) for 10 min. A substrate solution is then mixed with the LAL-sample and incubated at 37 °C (±1 °C) for an additional 6 min. The reaction was stopped with stop reagent. The level of endotoxin was determined by spectrophotometrically and calculated from a standard curve. The detection limit of this end point chromogenic assay was 0.01EU/ml. Samples with LPS level below the detection limit were taken as 0 EU/ml. We ran samples in duplicate and subtracted the background.

### Real-time 16S rDNA PCR

We use real-time 16S rDNA PCR described by Kramski, et al. [17] to quantify the possible bacteria in the blood of the patients. First, we prepare Master mix with shrimp nuclease (SNuc) treatment to eliminate bacterial double strand DNA in the environment. Cycling conditions for SNuc treatment of the master mix, 37 °C for 10 min followed by 72 °C for 20 min. We use forward primer (5′-AACAGGATTAGATACCCTGGTAG-3′) and reverse primer (5′ GGTTCTKCGCGTTGCWTC -3′) for PCR reaction. Cycling condition for PCR: 95 °C for 2 min, 40 cycles of 95 °C for 2 min, 95 °C for 5 s, and 60 °C for 30 s.

### Statistical analysis

We use SAS software (version 9.1) to analyze categorical variables by chi-square or Fisher’s exact test and continuous variables by the Wilcoxon’s rank sum test.

## Results

We recruited 60 ESRD patients (33 male and 27 female patients) with mean age 63.6 years who presented to emergency department (ED) from November 1, 2013 to October 31, 2014. Among them, 30 patients presented with ACS, and 30 patients without ACS.The later group included presentations of fever, abdominal pain, gastrointestinal tract bleeding, general malaise, and dizziness. Twenty-one patients had pre-existing coronary artery disease (CAD). Demographic and clinical information of ESRD patients presented to ED is shown in Tables 1 and 2.

**Table 1:** Comparison of continuous variables of demographic and clinical information between ESRD patients with and without acute coronary syndrome

	ACS group (*n* = 30)	Control group (*n* = 30)	p* value
Age	65.3 ± 11.6	62.6 ± 8.5	0.68
BMI (kg/m^2^)	26.2 ± 4.6	25.7 ± 3.4	0.50
Laboratory finding			
Prothrombin time (seconds)	11.60 (0.40)	12.10 (0.50)	0.18
INR	1.10 (0.10)	1.09 (0.10)	0.29
APTT (seconds)	36.10 (2.80)	36.40 (3.50)	0.31
Platelets (×10^3^/mm^3^)	265.42 (150.31)	271.26 (60.23)	0.51
CRP (mg/L)	25.70 (50.36)	51.39 (82.14)	0.1
Troponin-I (ng/mL)	1.14 (3.12)	0.05 (0.06)	<0.01

*p vale for Wilcoxon’s rank sum test

**Table 2:** Comparison of categorical variables of demographic and clinical information between ESRD patients with and without acute coronary syndrome

	ACS group (*n* = 30)	Control group (*n* = 30)	p* value
Sex			
Male	18	14	0.3
Clinical presentations			
Acute coronary syndrome	30	0	NA
Fever	0	11	NA
Abdominal pain	0	7	NA
Gastrointestinal tract bleeding	0	6	NA
General malaise	0	4	NA
Dizziness	0	2	NA
Past history			
Coronary artery disease	12	9	0.42
Congestive heart failure	1	3	0.30
Diabetes	11	17	0.12
Hypertension	21	16	0.18
Cerebrovascular accident	4	2	0.39
Malignancy	3	3	1
Gastrointestinal disease^a^	4	3	0.69
Recent infectious disease^b^	3	4	0.69

*p value for Fisher’s exact test

^a^Gastrointestinal disease includes hepatitis B and C, pancreatitis, peptic ulcers, and biliary tract stone

^b^Recent infectious disease includes pneumonia, bacteremia, cellulitis, arteriovenous fistula infection

**Fig. 1:**
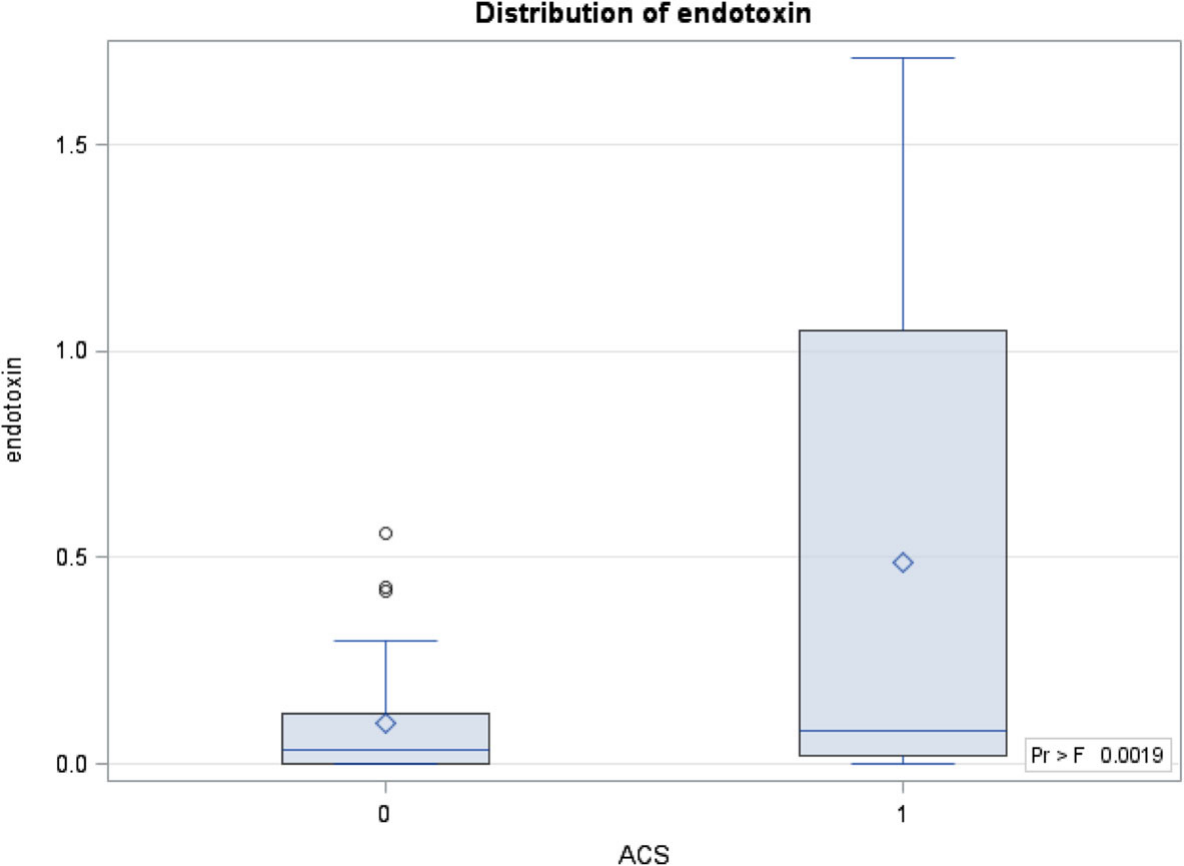
Comparision of serum endotoxin level between ESRD patients with and without ACS (*p* < 0.01). The boxes indicate median, 25th, and 75th percentile; whisker caps indicate 5th and 95th percentile; open circles indicate outliers

**Fig. 2:**
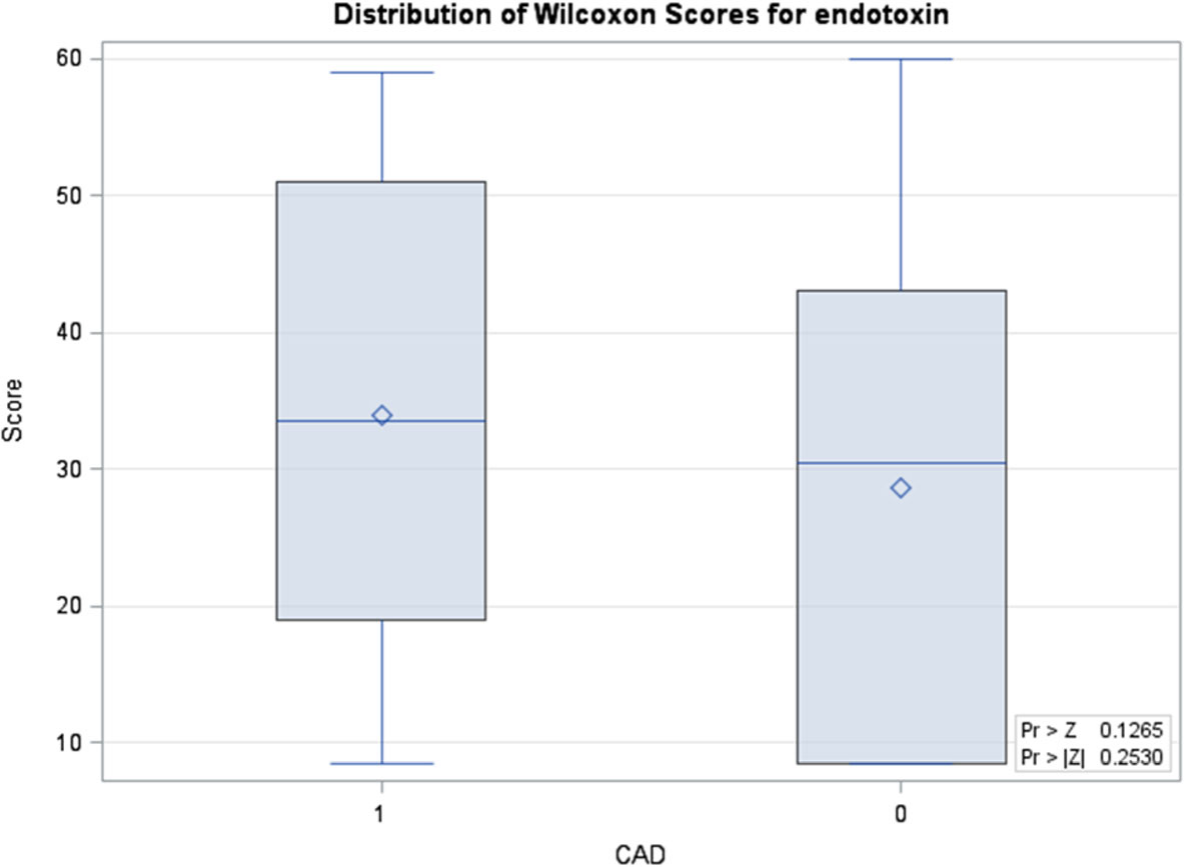
Comparision of serum endotoxin level between ESRD patients with and without pre-existing coronary artery disease (*p* = 0.13). The boxes indicate median, 25th, and 75th percentile; whisker caps indicate 5th and 95th percentile; open circles indicate outliers

**Fig. 3:**
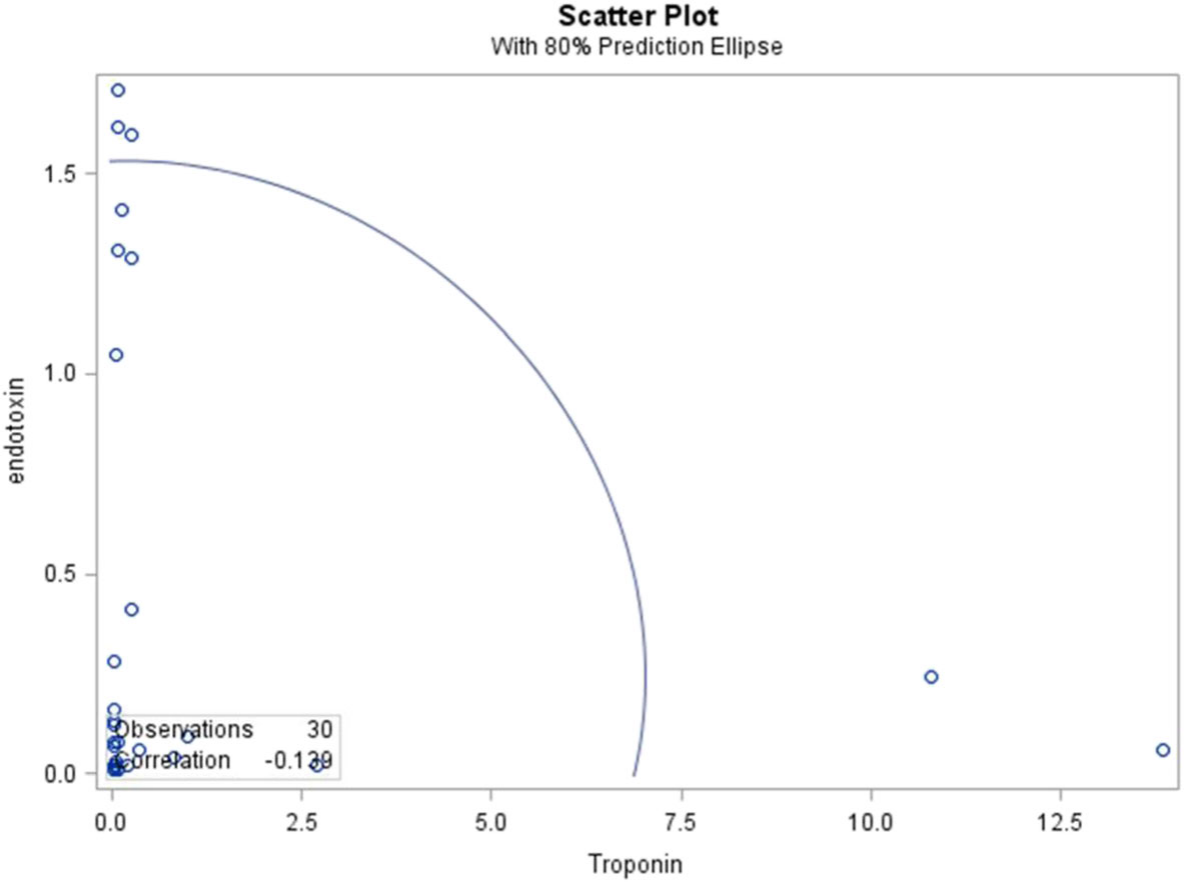
Scatter plot of correlation between entotoxin and troponin-I

The average endotoxin level of ESRD patients with ACS was 0.49 ± 0.12 EU/mL, which was significantly higher than that of ESRD patients without ACS (0.10 ± 0.08 EU/mL) (Table 1, Fig. 1). Systemic inflammatory marker C-reactive protein (CRP) was positively correlated with endotoxin (*r* = 0.574, *p* = 0.007). Among those patients with ACS, there was no significant difference in endotoxin level between ESRD patients with pre-existing CAD (0.45 ± 0.60 EU/mL) and without CAD (0.23 ± 0.44 EU/mL, *p* = 0.12) (Fig. 2). The average troponin-I level of ESRD patients with ACS was 1.14 ± 3.12 ng/mL, which was significantly higher than that of ESRD patients without ACS (0.05 ± 0.06 ng/mL). However, troponin-I level was not correlated with endotoxin level (*r* = −0.12) (Fig. 3).

Although endotoxin level was higher in ESRD patients with ACS, nucleotide sequences of bacteria were not detected in the serum by the real-time 16S rDNA PCR.

## Discussion

Previous study showed that endotoxin is related to systemic inflammation and atherosclerosis in CKD and peritoneal dialysis patients [18]. Chlamydial lipopolysaccharide is present in serum during acute coronary syndrome [19]. There is no study showing that endotoxin is related to ACS in ESRD patients. Our study showed that serum endotoxin level of the ESRD patients with ACS was significantly higher than that without ACS, indicating endotoxin may play an important role in ESRD patients with ACS.

One study showed that even a low endotoxin level contributes to the development of atherosclerosis [20]. Our data showed that endotoxin level of ESRD patients of ACS, regardless of pre-existing CAD, seemed to be high, however, there was no significant difference between these patients with and without pre-existing CAD (0.45 ± 0.60, 0.23 ± 0.44 EU/mL, individually, *p* = 0.12). This can be explained by the fact that the baseline of endotoxin level in ESRD patients remains high than health individuals. Chronic status of endotoxemia contributes to chronic inflammation and the development of atherosclerosis, which might be supported by our finding that endotoxin level was positively correlated with CRP. In contrast, acute stage of cardiovascular disease, such as ACS, is associated with high level of endotoxemia, suggesting that increase in endotoxin may accelerate atherosclerosis and acute coronary syndrome.

Troponin is a good indicator in the evaluation of the risk of myocardial muscle injury or acute coronary syndrome in the early stage [21]. Although elevated troponin-I is sometimes seen in symptoms free CKD patients [22, 23], in our study, troponin-I is significantly higher in ESRD patients with ACS than that in ESRD patients without ACS; Previous study showed that Endotoxin/LPS increased directly with troponin level in patients with periodontal disease [24]. However, in our study, endotoxin is not correlated with troponin-I level in these patients. There is no dose-response relationship between endotoxin and troponin-I. It may suggest that endotoxin does not reflect the magnitude of cardiac tissue damage, and endotoxin alone cannot predict the severity of ACS. The lack of association of endotoxin with troponin -I suggests that there was minimal effect on blood clotting or platelets.

Endotoxin derived from lipopolysaccharide (LPS) has been used as a surrogate of Gram negative bacteria translocation from gastrointestinal tract. However, this method is unable to detect microbial translocation of Gram positive bacteria in the intestine. We tried to search for the evidence of nucleotide sequences of any bacteria in the serum of ESRD patients. However, nucleotide sequences of bacteria were not detected in this study. It may be in part due to the methodological difficulty involved in obtaining direct samples of mesenteric lymph nodes or portal venous blood since we used serum sample to detect microbial nucleotide sequences. Alternatively, it may be explained by the result of transient bacteremia. There might be no microbes in the systemic blood stream while collecting blood of the patients. Serial blood samples might be used in the future study to improve the probability of detecting microbial nucleotides.

There were limitation in this study. We did not examine the periodontal health of the patients. Periodontal disease has been discussed as an important contributing factor to coronary artery disease. Future study for adjusting the effect of periodontal disease for CAD is needed. Secondly, we did not collect a randomized population. This makes correction for extraneous factors difficult, e.g., subclinical infection, and malnutrition.

## Conclusion

Our study shows that endotoxemia is associated with ESRD patients with ACS. An elevated endotoxin is likely due to translocation of Gram negative bacteria in the gut. However, endotoxin level is not correlated with troponin-I level, indicating endotoxin itself may not reflect the magnitude of cardiac tissue damage.

## Abbreviations

ACS: Acute coronary syndrome
CAD: Coronary artery disease
CKD: Chronic kidney disease
ED: Emergency department
ESRD: End-stage renal disease
LAL: Limulus amoebocyte lysat
LPS: Lipopolysaccharide
SNuc: Shrimp nuclease
